# Supplementary material for: Transcriptomic Adjustments in a Freshwater Ectoparasite Reveal the Role of Molecular Plasticity for Parasite Host Shift
Source: Genes (Basel). 2022 Mar 16;13(3):525. doi: 10.3390/genes13030525 (PMC8952325; doi:10.3390/genes13030525)
Supplement: Supplementary file 1 [file genes-13-00525-s001.zip › Supplementary_Material_Figure S1.pdf]

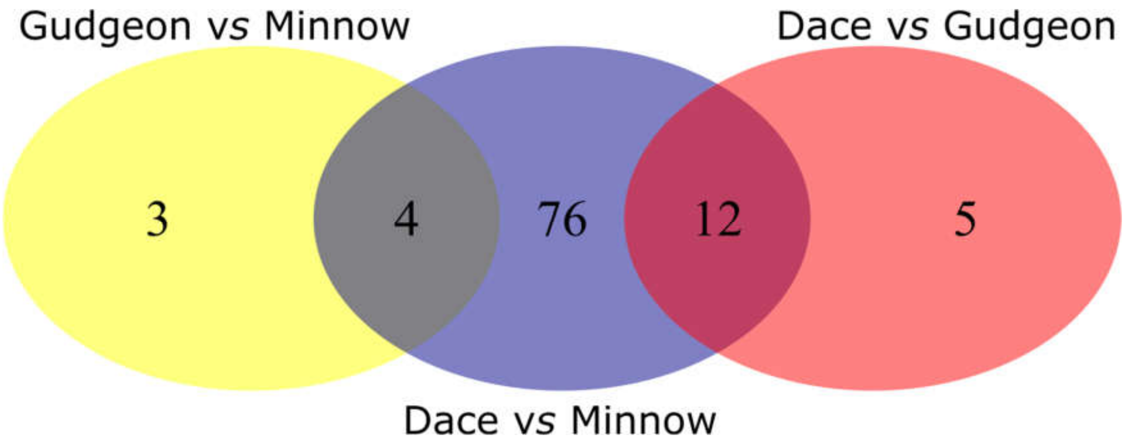

Figure S1. Venn diagram presenting the genes shared between the different pairwise comparisons between parasites exploiting different host species. Pairwise comparisons are named after the name of the host species on which T. polycolpus was collected (eg. Gudgeon vs. Minnow refers to the comparison between parasites sampled on gudgeon and parasites sampled on minnow).
