# Supplementary material for: Transcriptomic Adjustments in a Freshwater Ectoparasite Reveal the Role of Molecular Plasticity for Parasite Host Shift
Source: Genes (Basel). 2022 Mar 16;13(3):525. doi: 10.3390/genes13030525 (PMC8952325; doi:10.3390/genes13030525)
Supplement: Supplementary file 1 [file genes-13-00525-s001.zip › Supplementary_Material_Figure S2.pdf]

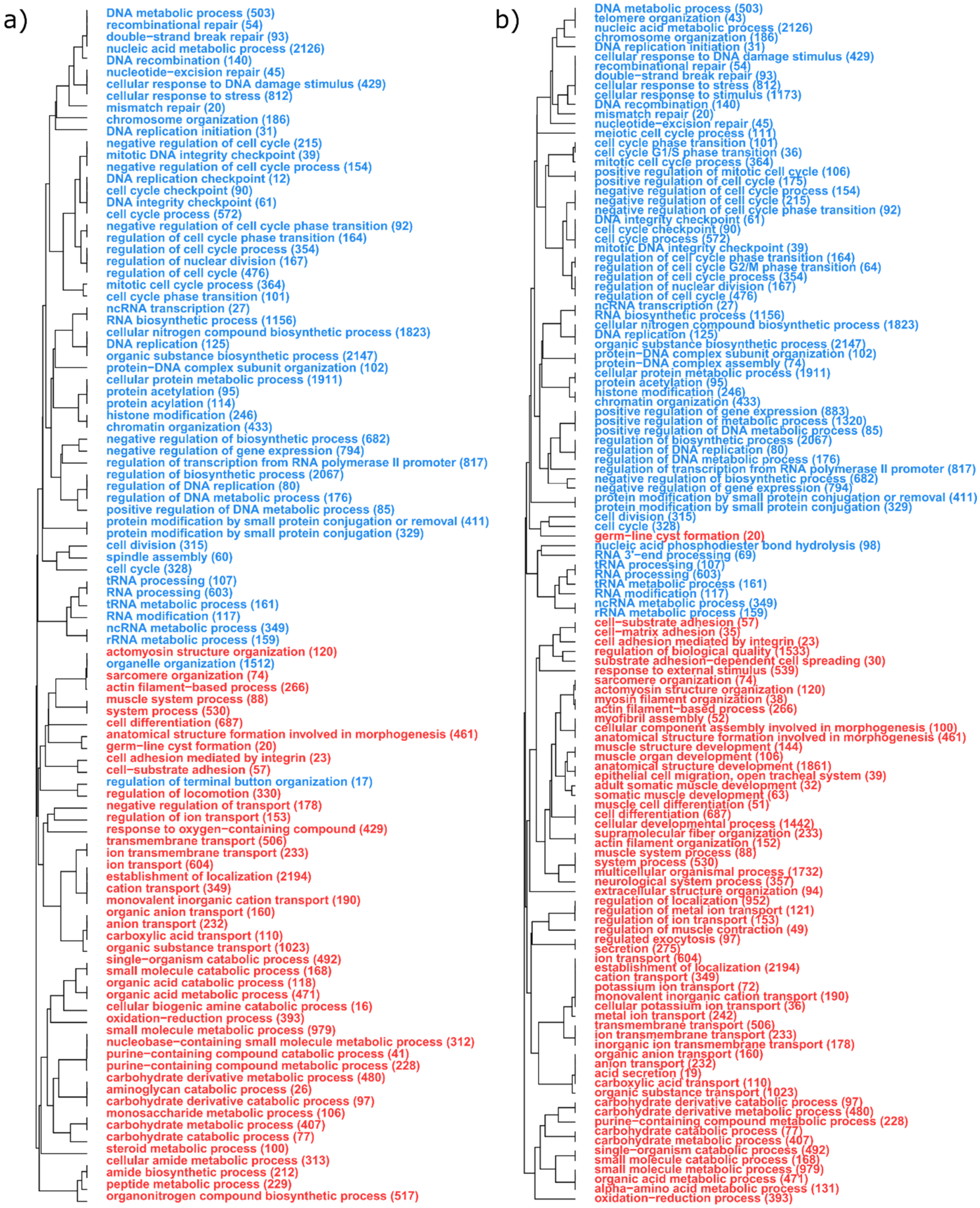

Figure S2. Gene ontologies (GOs) significantly enriched in differentially expressed genes (FDR < 0.001) for the comparison between (a) parasites infecting minnow and dace and (b) parasites infecting gudgeon and dace. Blue GOs refer to GOs enriched in under-expressed genes between alternative host parasites compared to principal host parasites, and red GOs refer to GOs enriched in over-expressed genes between alternative host parasites compared to principal host parasites. The total number of genes involved in each GO is given in brackets.
