# Supplementary material for: Transcriptomic Adjustments in a Freshwater Ectoparasite Reveal the Role of Molecular Plasticity for Parasite Host Shift
Source: Genes (Basel). 2022 Mar 16;13(3):525. doi: 10.3390/genes13030525 (PMC8952325; doi:10.3390/genes13030525)
Supplement: Supplementary file 1 [file genes-13-00525-s001.zip › Supplementary_Material_Figure S3.pdf]

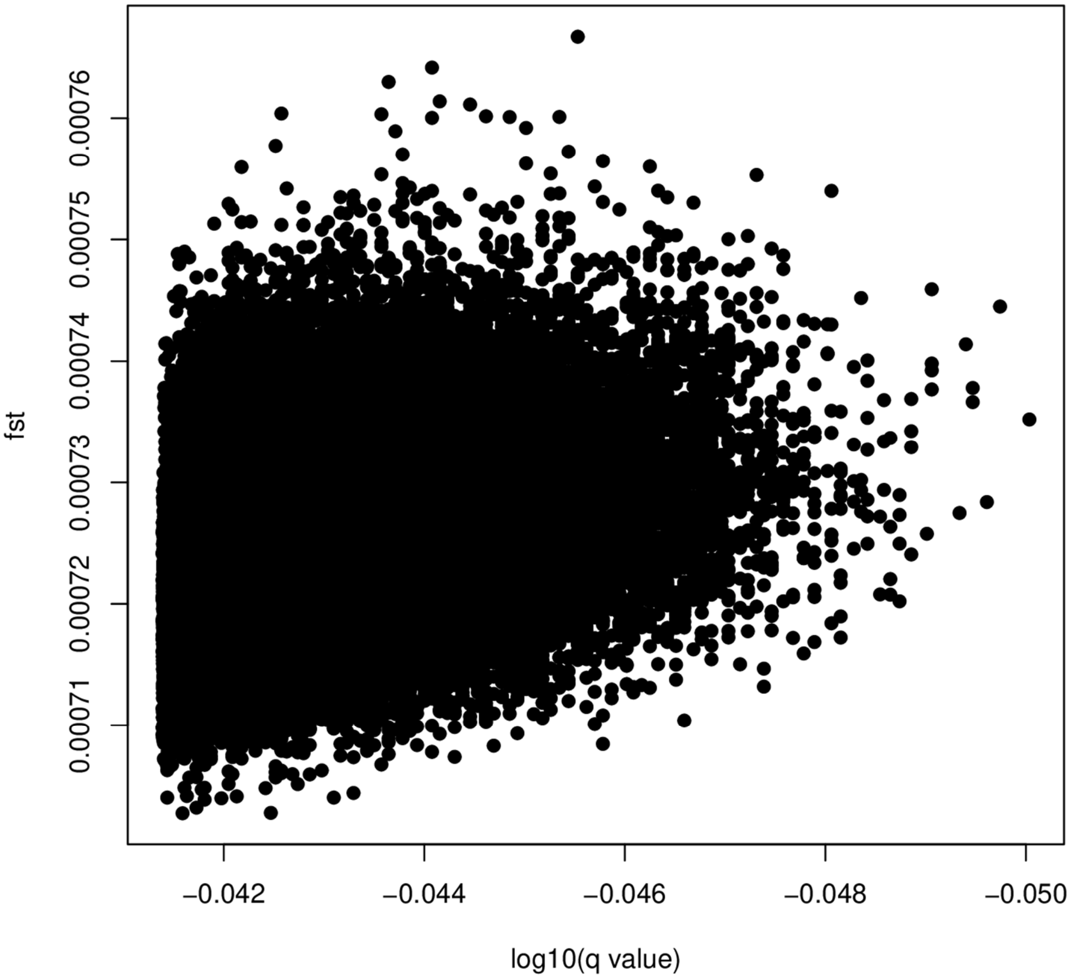

Figure S3. Plot showing Fst values and corresponding log transformed adjusted p-values for the 53 645 high-quality SNPs identified from T. polycolpus RNA-sequencing. Each dot represents a single SNP; none of them has been identified as a significant outlier.
