# Supplementary material for: Transcriptomic Adjustments in a Freshwater Ectoparasite Reveal the Role of Molecular Plasticity for Parasite Host Shift
Source: Genes (Basel). 2022 Mar 16;13(3):525. doi: 10.3390/genes13030525 (PMC8952325; doi:10.3390/genes13030525)
Supplement: Supplementary file 1 [file genes-13-00525-s001.zip › Supplementary_Material_File S2.pdf]

Supplementary File S2

Supplementary methods

Variant calling and FST outlier analysis

To investigate signature of selection between parasites collected from different host species we aimed to identify Single Nucleotide Polymorphism (SNPs) from RNA sequencing. To do so we used the Genome Analysis Toolkit (GATK version 4.0.1.2 McKenna *et al.*, 2010) and followed the recommendations regarding variant calling based on transcriptome assembly of non-model organisms presented in Eldem et al. (2017) (Eldem *et al.*, 2017). Succinctly, quality trimmed reads were first aligned on the *T. polycolpus* transcriptome using the aligner Bowtie2 using the same parametrization used for the transcript quantification (Langmead & Salzberg, 2012). Picard (version 2.1.1., http://picard.sourceforge.net/) and SAMtools (version 1.3.1, Li *et al.*, 2009) were then used to conduct the following pre-processing steps: 1) Assign a read group to each sample, 2) sort aligned reads, 3) remove duplicates and 4) merge '.bam' alignment results in fifteen ready reads files. After these pre-processing steps, we used the GATK tools to call potential variants. First, the fifteen ready reads files were parsed to *HaplotypeCaller* to call variants in each sample. Each genomic Variant Call Format (gVCF) files were then combined in one single gVCF file using the *CombineGVCFs*_function. The *GenotypeGVCFs* function was next used to perform the joint genotyping. Variants were filtered out when having a phred-scaled confidence threshold below 30. At this stage, since we were only interested in SNPs, we used the function *SelectVariant* to sort SNPs and stored them in a raw SNPs gVCF file. We then filtered the variants using the GATK hard filtering method (*VariantFiltration* function) and self-defined parameters (QD < 5.0, FS > 30.0, MQ < 10.0, MQRankSum < -12.5, ReadPosRankSum < -8.0) resulting in a set of 94 403 SNPs.

To detect variants under selection that could differentiate parasites from different host species we conducted an FST outlier analysis using the software Bayescan (version 2.0, Foll and Gaggiotti 2008). First, the script *make_bayescan_input.py* provided in De Wit et al. (2012) (De Wit *et al.*, 2012) was run to convert our set of SNPs in Bayescan format with the number of individual required per population set to 4 and resulting in a set

of 61 880 SNPs. We removed from this set of SNPs those having a low allelic frequency (i.e., minor alleles must be at least recorded twice otherwise they likely corresponds to sequencing errors) using the option –*d* in bayescan that allows discarding any listed SNPs. Finally, we run the FST outlier test on the resulting 53 645 high quality SNPs.

Supplementary results

Detection of selection

The outlier Fst analysis based on the 53 645 identified SNPs and aiming at comparing the level of genetic differentiation between parasites from the three host species revealed very low Fst values with an average of 0.001±.001 (±SE). No Fst outliers –and consequently no trace of selection– were identified among all the investigated protein-coding genes (Supplementary files, Fig S3, Supplementary Table S1).
